# Antinociceptive Activity of Petroleum Ether Fraction of* Clinacanthus nutans* Leaves Methanolic Extract: Roles of Nonopioid Pain Modulatory Systems and Potassium Channels

**DOI:** 10.1155/2019/6593125

**Published:** 2019-08-05

**Authors:** Zainul Amiruddin Zakaria, Mohammad Hafiz Abdul Rahim, Rushduddin Al Jufri Roosli, Mohd Hijaz Mohd Sani, Najihah Hanisah Marmaya, Maizatul Hasyima Omar, Lay Kek Teh, Mohd. Zaki Salleh

**Affiliations:** ^1^Department of Biomedical Science, Faculty of Medicine and Health Science, Universiti Putra Malaysia, 43400 Serdang, Selangor, Malaysia; ^2^Integrative Pharmacogenomics Institute (iPROMISE), Level 7, FF3, Faculty of Pharmacy, Universiti Teknologi MARA, Puncak Alam Campus, 42300 Bandar Puncak Alam, Selangor, Malaysia; ^3^Department of Biomedical Sciences and Therapeutics, Faculty of Medicine and Health Science, Universiti Malaysia Sabah, 88400 Kota Kinabalu, Sabah, Malaysia; ^4^Faculty of Business and Management, Universiti Teknologi MARA, Melaka Campus, 75300, Melaka, Malaysia; ^5^Phytochemistry Unit, Herbal Medicine Research Centre, Institute for Medical Research, Jalan Pahang, 50588 Kuala Lumpur, Malaysia

## Abstract

Methanolic extract of* Clinacanthus nutans* Lindau leaves (MECN) has been reported to exert antinociceptive activity. The present study aimed to elucidate the possible antinociceptive mechanisms of a lipid-soluble fraction of MECN, which was obtained after sequential extraction in petroleum ether. The petroleum ether fraction of* C. nutans* (PECN), administered orally to mice, was (i) subjected to capsaicin-, glutamate-, phorbol 12-myristate 13-acetate-, bradykinin-induced nociception model; (ii) prechallenged (intraperitoneal (i.p.)) with 0.15 mg/kg yohimbine, 1 mg/kg pindolol, 3 mg/kg caffeine, 0.2 mg/kg haloperidol, or 10 mg/kg atropine, which were the respective antagonist of *α*_2_-adrenergic, *β*-adrenergic, adenosinergic, dopaminergic, or muscarinic receptors; and (iii) prechallenged (i.p.) with 10 mg/kg glibenclamide, 0.04 mg/kg apamin, 0.02 mg/kg charybdotoxin, or 4 mg/kg tetraethylammonium chloride, which were the respective inhibitor of ATP sensitive-, small conductance Ca^2+^-activated-, large conductance Ca^2+^-activated-, or nonselective voltage-activated-K^+^ channel. Results obtained demonstrated that PECN (100, 250, and 500 mg/kg) significantly (P<0.05) inhibited all models of nociception described earlier. The antinociceptive activity of 500 mg/kg PECN was significantly (P<0.05) attenuated when prechallenged with all antagonists or K^+^ channel blockers. However, only pretreatment with apamin and charybdotoxin caused full inhibition of PECN-induced antinociception. The rest of the K^+^ channel blockers and all antagonists caused only partial inhibition of PECN antinociception, respectively. Analyses on PECN's phytoconstituents revealed the presence of antinociceptive-bearing bioactive compounds of volatile (i.e., derivatives of *γ*–tocopherol, *α*–tocopherol, and lupeol) and nonvolatile (i.e., cinnamic acid) nature. In conclusion, PECN exerts a non-opioid-mediated antinociceptive activity involving mainly activation of adenosinergic and cholinergic receptors or small- and large-conductance Ca^2+^-activated-K^+^ channels.

## 1. Introduction

Pain, an unpleasant physical and emotional experience, comes in various forms: acute, chronic, visceral, inflammatory, or neuropathic [1]. Current medications used to treat pain are divided into opioids (i.e., morphine and fentanyl) and nonopioids (i.e., nonsteroidal anti-inflammatory drugs (NSAIDs) (i.e., aspirin and ibuprofen), acetaminophen; antidepressants; and antiseizures) [2]. Despite the significant advances in the development of synthetic drugs and the availability of those drugs in the market as prescribed and nonprescribed drugs, their effectiveness has been surpassed by the mild to serious adverse effects ranging from gastric discomfort to addiction [3]. Moreover, the increase in life expectancy and persistent pathologies in conjunction with it are anticipated to increase the incidence of accompanying pain annually especially in elderly patients [4]. Considering the fact that the elderly require a more sensitive treatment, new therapeutic agents with increased efficacy, fewer side effects, and lower costs and an improved quality of life should be one of the primary objectives in modern medical research [1].

Medicinal plants are excellent sources for new drugs and may provide valuable therapeutic alternatives. They are accessible, affordable, and culturally appropriate sources of primary health care for more than 80 percent of Asia population [5]. Many people in the developing countries especially those living in the rural area, with low income, depend on medicinal plants as the principal means of preventing and curing illnesses. In many developing countries, the use of plants as analgesic was mainly as folk medicine based on ancient tradition [6]. Interestingly, natural pain treatments using herbal medicine have recently gained popularity worldwide because of their natural origin and fewer side effects. Other than being effective against pain and possessing less or no adverse effects in comparison to the existing drugs, medicinal plants may be more affordable to the people with lower economic background.

One of the medicinal plants being studied for its potential to attenuate pain is* Clinacanthus nutans* Lindau. Known to the Malay as “*Belalai Gajah,*”* C. nutans* is a small shrub that belongs to the family Acanthaceae and commonly grows in tropical Southeast Asian countries, including Malaysia. The plant is traditionally used to treat various ailments including pain [7, 8] and scientifically proven to possess various pharmacological activities, including an ability to attenuate nociceptive response [9, 10]. Using methanol extract of* C. nutans* (MECN), Abdul Rahim et al. [9] demonstrated the peripherally- and centrally-mediated antinociceptive activity of MECN, which involved the modulation of opioid receptor system and nitric oxide- (NO-) mediated/cyclic guanosine monophosphate- (cGMP-) independent pathway. Further study by Zakaria et al. [10] revealed that MECN exerted an antinociceptive activity, which was reversed upon (i) inhibition of the opioidergic, *α*_2_-noradrenergic, *β*-adrenergic, adenosinergic, dopaminergic, and cholinergic receptors; (ii) opening of various K^+^ channels, namely, the voltage-activated-, Ca^2+^-activated-, and ATP-sensitive-K^+^ channels; and (iii) modulation of protein kinase C (PKC)-, bradykinin-, transient receptor potential cation channel subfamily V member 1 (TRPV1)-, and glutamatergic-signaling pathways. In support of these findings, the phytochemical analysis of MECN using HPLC-ESI had identified at least sixteen compounds, namely, gallic acid, 4-hydroxybenzoic acid, caffeic acid, coumaric acid, ferulic acid, schaftoside, vitexin, orientin, isoorientin, isovitexin, luteolin, apigenin, forsythosides H, forsythosides I, diosmetin glycoside, and diosmetin. Of these, at least gallic acid, caffeic acid, ferulic acid, vitexin, and apigenin have been reported to exert antinociceptive activity when given orally [9].

With the antinociceptive potential of MECN proven as described above, further study was designed with an attempt to separate the bioactive compounds in MECN based on their increasing polarity using three types of solvents, namely, petroleum ether, ethyl acetate, and water. In the preliminary study, petroleum ether partition of MECN (PECN) was found to exert the most effective antinociceptive effect when assessed using the abdominal writhing test and, thus, was selected for further antinociceptive investigation. PECN was also found to inhibit nociceptive response in the hot plate test and formalin-induced paw licking test. In terms of mechanisms of action involved, the antinociceptive activity of PECN was also found to be modulated via the opioid receptor system and the NO-mediated/cGMP-independent pathway as seen with MECN (Zakaria, 2019;* personal communication*). Based on the recent findings, further investigations were carried out to determine the mechanisms of antinociception of PECN by focusing on the involvement of various nonopioid receptor systems and different types of potassium channels.

## 2. Materials and Methods

### 2.1. Plant Collection

The leaves of* C. nutans* were donated by Clinnthus Enterprise (Kuala Lumpur, Malaysia) and a voucher specimen (No. SK 2679/15) had been deposited in the herbarium of the Institute of Bioscience (IBS), Universiti Putra Malaysia (UPM), Serdang, Selangor, Malaysia.

### 2.2. Preparation of PECN

The preparation of MECN was described in detail in our previous published study [9] while the procedures involved in the preparation of semipurified petroleum ether partition were described in detail by Zakaria et al. [11].

### 2.3. Experimental Animals

Adult male ICR mice (25-30 g) were used in the present study. The animals were cared for, handled, and provided housing facilities according to the detailed procedures described by Abdul Rahim et al. [9]. All experimental animals were cared for and treated according to the ethical guidelines adopted by UPM Institutional Animal Care and Use Committee (Ref. Number UPM/IACUC/AUP-R032/2013). The quantity of animals and concentrations of noxious stimuli used were the minimum needed to establish the consistent effects of the treatments. Experiments were carried out between 09:30 and 18:30 h to lessen the effects of environmental changes.

### 2.4. Drugs and Chemicals

The following drugs, acetylsalicylic acid (ASA), capsaicin, capsazepine (CAPZ), l-glutamic acid, phorbol 12-myristate 13-acetate (PMA), bradykinin, yohimbine, pindolol, caffeine, haloperidol, atropine, glibenclamide, apamin, charybdotoxin, tetraethylammonium chloride were procured from Sigma-Aldrich (St. Louis, MO, USA). Acetic acid and dimethyl sulfoxide (DMSO) were procured from Fisher Scientific (Fair Lawn, NJ, USA). All drugs (i.e., bradykinin, capsaicin, l-glutamic acid, and PMA) were dissolved in physiological saline (0.9% [w/v] NaCl), while PECN, ASA, and CAPZ were dissolved in 10% DMSO (v/v). The vehicle had no effects* per se* on the nociceptive responses in mice when administered alone. The other solutions (i.e., 0.6% acetic acid) were prepared in 0.9% NaCl. All drugs and chemicals were freshly prepared prior to use and administered in the volume of 10 mL/kg.

### 2.5. Determination of the Possible Involvement of Non-Opioid-Mediated Mechanisms in the Modulation of Antinociceptive Activity of PECN

The assays used to determine the involvement of non-opioid-mediated nociceptive systems in the modulation of PECN-induced antinociceptive activity included the capsaicin-, glutamate-, phorbol 12-myristate 13-acetate (PMA), and bradykinin-induced paw licking nociceptive assays [10].

#### 2.5.1. Determination of the Antinociceptive Effect of PECN on Capsaicin-Induced Paw Licking Nociception

For the capsaicin-induced paw licking nociception assay, five groups of mice (*n *=* 6*) were treated with vehicle (10 mL/kg, p.o.), CAPZ (TRPV1 receptor antagonist; 0.17 mmol/kg, p.o.; served as the positive control); or PECN (100, 250, 500 mg/kg, p.o.), respectively. Sixty minutes after the administration of each test solution, the animals were injected [20 *μ*L; intraplantar (i.pl)] with capsaicin (1.6 *μ*g/paw) into the ventral surface of the right hind paw. Immediately after the administration of capsaicin, the animals were individually placed in the transparent glass cage observation chamber from 0 to 5 min and the amount of time the mice spent licking on the injected paw (an indicator of nociception) was recorded utilizing a chronometer.

#### 2.5.2. Determination of the Antinociceptive Effect of PECN on Glutamate-Induced Paw Licking Nociception

For the glutamate-induced paw licking nociception assay, five groups of mice (*n *=* 6*) were treated with vehicle (10 mL/kg, p.o.), ASA (100 mg/kg; served as the positive control), or PECN (100, 250, 500 mg/kg, p.o.), respectively. Sixty minutes after the administration of each test solution, the animals were injected (20 *μ*L; i.pl) with glutamate (10 umol/paw) into the ventral surface of the right hind paw. The amount of time the mice spent licking on the injected paw was recorded from 0 to 15 min immediately after the administration of glutamate as described above.

#### 2.5.3. Determination of the Antinociceptive Effect of PECN on PMA-Induced Paw Licking Nociception

For the PMA-induced paw licking nociception assay, five groups of mice (*n *=* 6*) were treated with vehicle (10 mL/kg, p.o.), ASA (100 mg/kg; served as the positive control), or PECN (100, 250, 500 mg/kg, p.o.), respectively. Sixty minutes after the administration of each test solution, the animals were injected (20 *μ*L; i.pl) with PMA (a protein kinase C activator; 0.05 *μ*g/paw) into the ventral surface of the right hind paw. The amount of time the mice spent licking on the injected paw was recorded from 15 to 45 min immediately after the administration of PMA as described above.

#### 2.5.4. Determination of the Antinociceptive Effect of PECN on Bradykinin-Induced Paw Licking Nociception

For the bradykinin-induced paw licking nociception assay, five groups of mice (*n *=* 6*) were treated with vehicle (10 mL/kg, p.o.), ASA (100 mg/kg; served as the positive control), or PECN (100, 250, 500 mg/kg, p.o.), respectively. Sixty minutes after the administration of each test solution, the animals were injected (20 *μ*L; i.pl) with bradykinin (10 nmol/paw) into the ventral surface of the right hind paw. The amount of time the mice spent licking on the injected paw was recorded from 0 to 10 min immediately after the administration of bradykinin as described above.

#### 2.5.5. Determination on the Antinociceptive Effect of PECN Prechallenged with Various Receptor Antagonists and Assessed Using the Abdominal Writhing Test

The probable role of various nonopioid receptors in the modulation of PECN-induced antinociceptive activity was assessed as previously described by Zakaria et al. [10]. Briefly, the mice (*n = 6*) were pretreated (intraperitoneally; i.p.) with yohimbine (YOH; 0.15 mg/kg), pindolol (PDL; 1 mg/kg), caffeine (CAF; 3 mg/kg), haloperidol (HAL; 0.2 mg/kg), or atropine (ATR; 10 mg/kg) for 15 min before the administration (p.o.) of vehicle or 500 mg/kg PECN. Sixty minutes later, the animals were subjected to the abdominal writhing test via the injection (i.p.) of 0.6% acetic acid (10 mL/kg; a phlogistic agent). The number of abdominal writhing produced was counted cumulatively for 25 min starting 5 min after the acetic acid administration [10].

#### 2.5.6. Determination on the Antinociceptive Effect of PECN Prechallenged with Different Types of Potassium Channels' Blocker and Assessed Using the Abdominal Writhing Test

To explore the possible involvement of several types of potassium channels in the modulation of antinociceptive activity of PECN, nine groups of mice (*n = 6*) were pretreated (i.p.) for 15 min with glibenclamide (GLIB; 10 mg/kg), apamin (APA; 0.04 mg/kg), charybdotoxin (CHAR; 0.02 mg/kg), or tetraethylammonium chloride (TEA; 4 mg/kg), which were the respective inhibitor of ATP sensitive K^+^ channel inhibitor, small conductance Ca^2+^–activated K^+^ channels, large conductance Ca^2+^–activated K^+^ channels, and nonselective voltage-dependent K^+^ channel, followed by the administration (p.o.) of either vehicle or 500 mg/kg PECN. The dose of each potassium channel blocker was selected based on previous report [12]. Sixty minutes later, the mice were subjected to the acetic acid-induced abdominal writhing test as described by Zakaria et al. [10].

### 2.6. Determination of PECN Phytoconstituents

#### 2.6.1. Phytochemical Screening of PECN

The phytochemical screening of PECN was performed according to the conventional protocols adapted from the study by Mamat et al. [13].

#### 2.6.2. HPLC Analysis of PECN

The HPLC analysis was conducted at the Laboratory of Phytomedicine, Forest Research Institute of Malaysia, Kepong, Malaysia, according to the protocols described by Mahmood et al. [14] but with slight modifications. The exact procedures were described in detail by Zakaria et al. [11].

#### 2.6.3. GC-MS Analysis of PECN

The GC-MS analysis of PECN was carried out using the same method that was used to determine the GC-MS profile of MECN [9].

### 2.7. Data Analysis

For the data analysis, GraphPad Prism version 6.04 for Windows (GraphPad Software, San Diego, CA, USA) was used. Data are expressed as the mean ± standard deviation (SD). The mean differences between the control and treatment groups were determined by using the one–way analysis of variance (ANOVA) followed by Bonferroni's post-test. In all cases, the differences were considered significant if* p *< 0.05.

## 3. Results

Based on the earlier study, PECN exerted significant antinociceptive activity: (i) at all doses (100, 250, and 500 mg/kg) in a dose-dependent manner when assessed using the acetic acid-induced abdominal writhing test; (ii) at 250 and 500 mg/kg when assessed using the hot plate test; and (iii) at 250 and 500 mg/kg in the first phase and at 100-500 mg/kg in the second phase of formalin-induced paw licking test.

### 3.1. Effect of PECN on Capsaicin-, Glutamate-, Phorbol 12-Myristate 13-Acetate (PMA)-, Bradykinin-Induced Paw Licking Nociception

**Figure 1:**
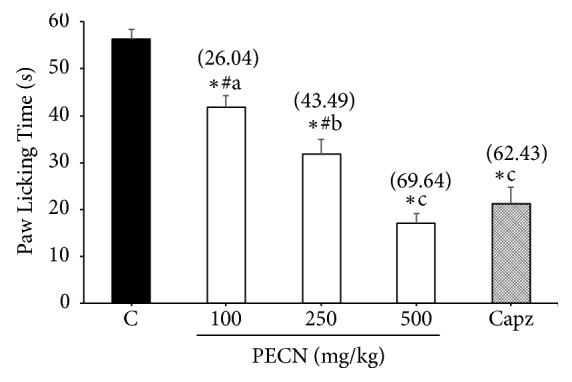
Effect of PECN on capsaicin–induced nociception in mice. Animals were treated with vehicle (10 mL/kg, p.o.), CAPZ (0.17 mmol/kg, p.o.), or PECN (100, 250, 500 mg/kg, p.o.) 60 min before intraplantar administration of capsaicin (1.6 *μ*g/paw prepared in normal saline; 20 *μ*L) into the right hind paw. Each column represents the mean ± SD of six mice. Statistical analyses were performed using 1–way ANOVA followed by Bonferroni's post hoc test. ^*∗*^*p* < 0.05 significantly differed when compared to control group; ^#^*p* < 0.05 significantly differed when compared to CAPZ; ^abc^*p* < 0.05 data with different superscript differed significantly when compared together. Values in parentheses denote percentage of inhibition.

Figure 1 depicted the antinociceptive profile of PECN on capsaicin-induced nociception in mice. PECN (100-500 mg/kg, p.o.) produced significant (*F*[4,25] = 15.53,* p*< 0.001) and dose-related inhibition of the capsaicin-induced neurogenic pain. At the tested doses, PECN reduced the paw-licking response by 26.04%, 43.49%, and 69.64%, respectively, compared with the control group. CAPZ (0.17 mmol/kg) which was used as a positive control drug showed 62.43% inhibition compared with the control group.

**Figure 2:**
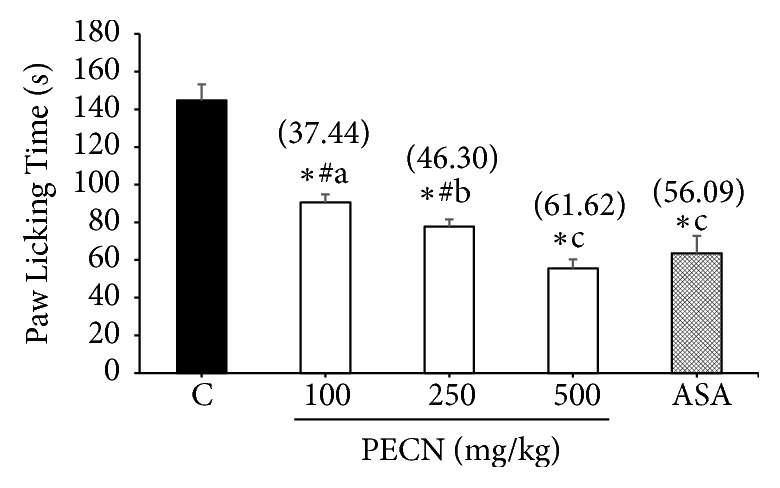
Effect of PECN on glutamate–induced nociception in mice. Animals were treated with vehicle (10 mL/kg, p.o.), ASA (100 mg/kg, p.o.), or PECN (100, 250, 500 mg/kg, p.o.) 60 min before intraplantar administration of glutamate (10 umol/paw prepared in normal saline; 20 *μ*L) into the right hind paw. Each column represents the mean ± SD of six mice. Statistical analyses were performed using 1–way ANOVA followed by Bonferroni's post hoc test. ^*∗*^*p* < 0.05 significantly differed when compared to control group; ^#^*p* < 0.05 significantly differed when compared to ASA; ^abc^*p* < 0.05 data with different superscript differed significantly when compared together. Values in parentheses denote percentage of inhibition.

The results provided in Figure 2 show that PECN (100-500 mg/kg, p.o.) produced significant (*F*[4,25] = 13.79,* p*< 0.001) and dose-related inhibition of glutamate-induced nociception with the percentage of inhibition observed of 37.44%, 46.30%, and 61.62%, respectively, compared with the control group. ASA (100 mg/kg) which was used as a positive control drug showed 56.09% inhibition compared with the control group.

**Figure 3:**
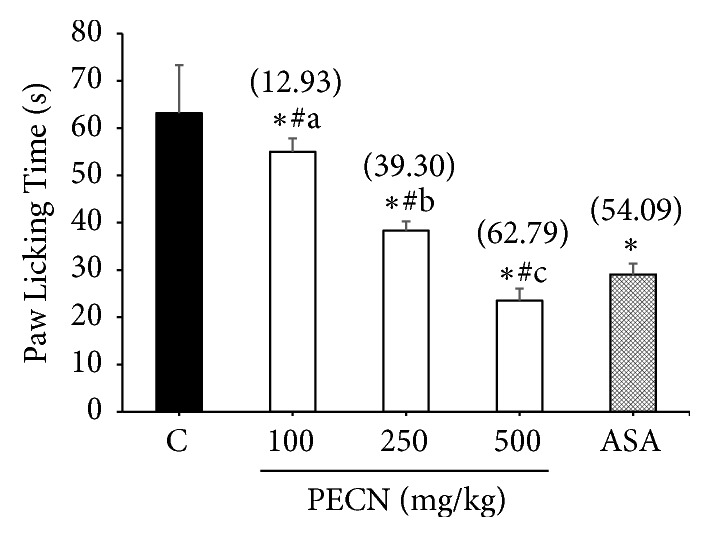
Effect of PECN on PMA–induced nociception in mice. Animals were treated with vehicle (10 mL/kg, p.o.), ASA (100 mg/kg, p.o.), or PECN (100, 250, 500 mg/kg, p.o.) 60 min before intraplantar administration of glutamate (0.05 *μ*g/paw prepared in normal saline; 20 *μ*L) into the right hind paw. Each column represents the mean ± SD of six mice. Statistical analyses were performed using 1–way ANOVA followed by Bonferroni's post hoc test. ^*∗*^*p* < 0.05 significantly differed when compared to control group; ^#^*p* < 0.05 significantly differed when compared to ASA; ^abc^*p* < 0.05 data with different superscript differed significantly when compared together. Values in parentheses denote percentage of inhibition.

As shown in Figure 3, PECN (100-500 mg/kg, p.o.) also produced a marked (*F*[4,25] = 15.29,* p*< 0.001) and dose-dependent inhibition of PMA-induced paw licking in mice with the percentage of inhibition observed of 12.93%, 39.30%, and 62.79%, respectively, compared with the control group. ASA (100 mg/kg) which was used as the positive control drug produced 54.09% of inhibition against PMA-induced nociception.

**Figure 4:**
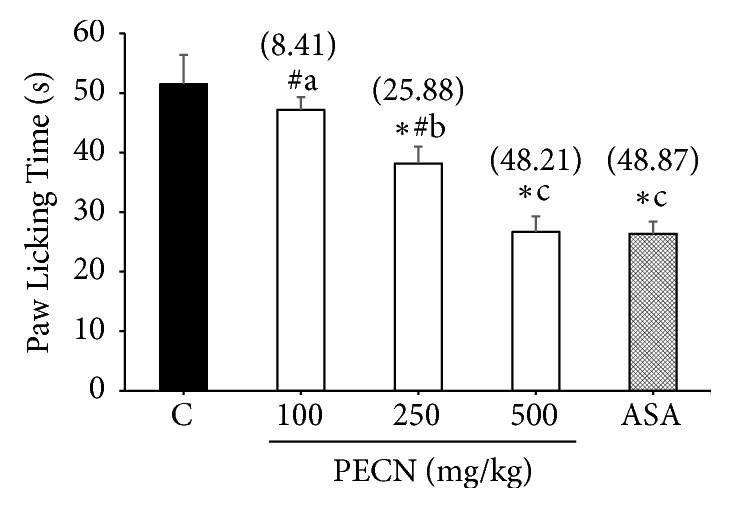
Effect of PECN on bradykinin–induced nociception in mice. Animals were treated with vehicle (10 mL/kg, p.o.), ASA (100 mg/kg, p.o.), or PECN (100, 250, 500 mg/kg, p.o.) 60 min before intraplantar administration of bradykinin (10 nmol/paw prepared in normal saline; 20 *μ*L) into the right hind paw. Each column represents the mean ± SD of six mice. Statistical analyses were performed using 1–way ANOVA followed by Bonferroni's post hoc test. ^*∗*^*p* < 0.05 significantly differed when compared to control group; ^#^*p* < 0.05 significantly differed when compared to ASA; ^abc^*p* < 0.05 data with different superscript differed significantly when compared together. Values in parentheses denote percentage of inhibition.

Other than that, the results depicted in Figure 4 show that PECN produced a marked (*F*[4,25] = 12.42,* p*< 0.001) and dose-dependent manner on the nociceptive caused by the i.pl injection of bradykinin in mice. Interestingly, only 250 and 500 mg/kg PECN show significant inhibition (*p*< 0.001) of bradykinin-induced nociception with the recorded reduction in paw-licking response of 25.88% and 48.21%, respectively, when compared against the control group. Meanwhile, PECN at 100 mg/kg reduced the paw-licking response by 8.41% when compared with the control group, although it did not show significant changes in nociception induced by bradykinin. Under similar conditions, ASA (100 mg/kg), used as the reference drug, produced 48.87% of inhibition against bradykinin-induced nociception.

### 3.2. Effect of PECN Prechallenged with Various Receptor Antagonists Assessed Using the Acetic Acid-Induced Abdominal Writhing Test

**Figure 5:**
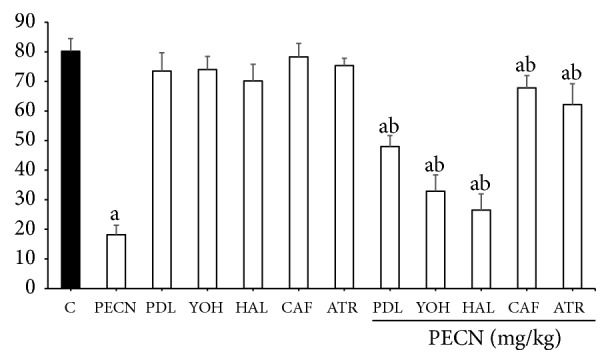
The involvement of various nonopioid receptor antagonists on PECN–induced antinociception in the acetic acid–induced abdominal writhing test in mice. Pindolol (PDL; 1 mg/kg, i.p.), yohimbine (YOH; 0.15 mg/kg, i.p.), Haloperidol (HAL; 0.2 mg/kg; i.p.), caffeine (CAF; 3 mg/kg, i.p.), or atropine (10 mg/kg, i.p.) was administrated 15 min before vehicle (10 mL/kg, p.o.) or PECN (500 mg/kg, p.o.). Each column represents the mean ± SD of six mice. Statistical analyses were performed using 1–way ANOVA followed by Bonferroni's post hoc test. ^a^*p* < 0.001 significantly differed when compared to control group. ^b^*p* < 0.001 significantly differed when compared to 500 mg/kg PECN–treated group.

As shown in Figure 5, pretreatment with *α*_2_-adrenoreceptor antagonist (0.15 mg/kg YOH; i.p.), *β*-adrenoreceptor, and 5-HT_1_ serotonergic antagonist (1 mg/kg PDL; i.p.), the nonselective adenosinergic receptor antagonist (3 mg/kg CAF; i.p.), the nonselective dopaminergic system antagonist (0.2 mg/kg HAL; i.p.), and the muscarinic cholinergic antagonist (10 mg/kg ATR; i.p.) significantly reversed (*p*< 0.001) the PECN-induced antinociception (500 mg/kg, p.o.) against acetic acid-induced abdominal writhing in mice, respectively. Each of the antagonists caused only partial inhibition of the antinociceptive activity exerted by PECN.

### 3.3. Involvement of Potassium Channels in the Modulation of PECN-Induced Antinociceptive Activity Assessed Using the Acetic Acid-Induced Abdominal Writhing Test

**Figure 6:**
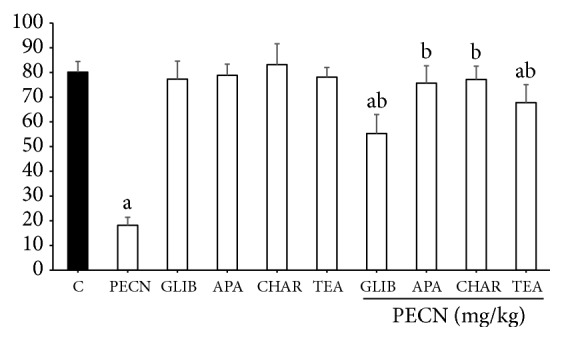
Effect of glibenclamide, apamin, charybdotoxin, and tetraethylammonium chloride on PECN–induced antinociception in the acetic acid–induced abdominal writhing test in mice. Animals were pretreated with glibenclamide (GLIB; 10 mg/kg, i.p.), apamin (APA; 0.04 mg/kg, i.p.), charybdotoxin (CHAR; 0.02, i.p.), or tetraethylammonium chloride (TEA; 4 mg/kg, i.p.) 15 min before oral administration of either vehicle (10 mL/kg) or PECN (500 mg/kg). Each column represents the mean ± SD of six mice. Statistical analyses were performed using 1–way ANOVA followed by Bonferroni's post hoc test. ^a^*p* < 0.001 significantly differed when compared to control group. ^b^*p* < 0.001 significantly differed when compared to 500 mg/kg PECN–treated group.

The pretreatment with ATP–sensitive K^+^ channel inhibitor GLIB (10 mg/kg, i.p.), inhibitor of small conductance Ca^2+^-activated K^+^ channels APA (0.04 mg/kg, i.p.), inhibitor of large conductance Ca^2+^-activated K^+^ channels CHAR (0.02 mg/kg, i.p.), and nonselective voltage-dependent K^+^ channel inhibitor TEA (4 mg/kg, i.p.) significantly reversed (*p*< 0.001) the antinociceptive effect of PECN (500 mg/kg, p.o.) in the acetic acid-induced abdominal writhing test, as shown in Figure 6. However, only APA and CHAR caused full inhibition of antinociceptive activity induced by PECN.

### 3.4. Phytochemical Groups of PECN

The PECN revealed the presence of flavonoids, saponins, steroids, and triterpenes and the absence of alkaloids and tannins.

### 3.5. Phytoconstituents of PECN following the HPLC Analysis

**Figure 7:**
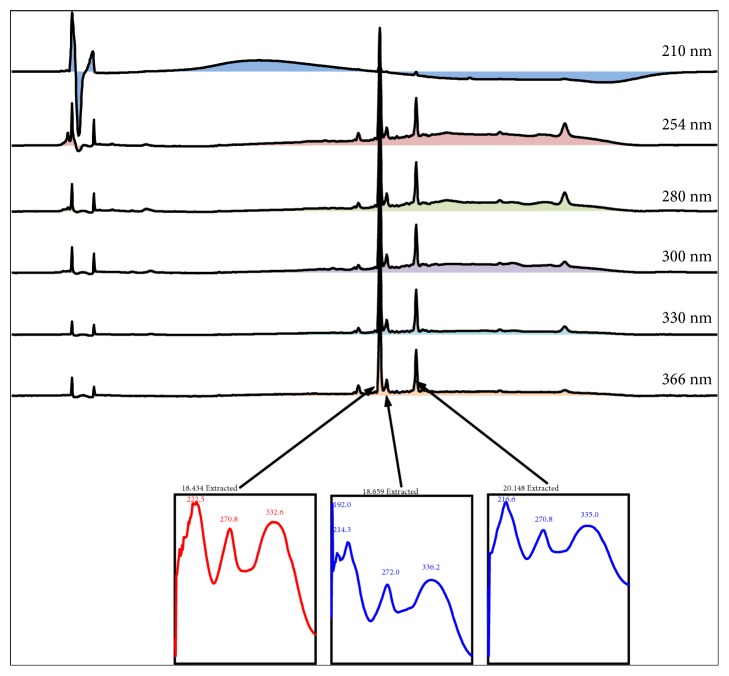
The UV spectra analysis of PECN. The UV spectra demonstrated the presence of 3 major peaks labelled as P1 (RT=18.434 min), P2 (RT=18.659 min), and P3 (RT=20.148 min), which were observed at their respective *λ*_max_ at the region of 222.5–332.6, 192.0–336.2, and 216.6–335.0 nm, respectively, suggesting, in part, the presence of phenylchroman (C_6_–C_3_–C_6_) skeletal structures.

**Figure 8:**
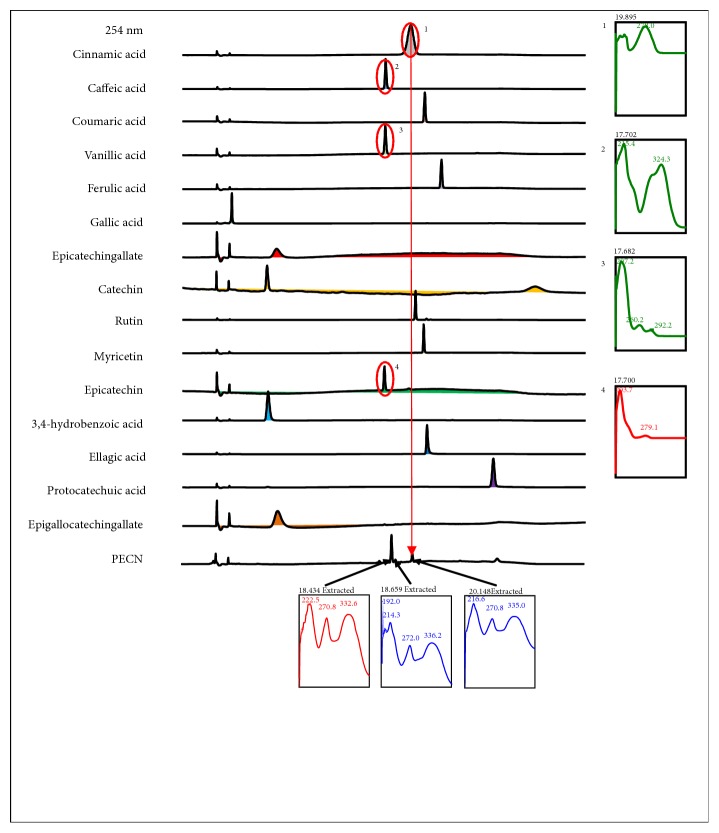
Comparison between chromatograms of the several standard compounds, namely, cinnamic acid, caffeic acid, coumaric acid, vanillic acid, ferulic acid, gallic acid, epicatechin gallate, catechin, rutin, myricetin, epicatechin, 3,4–hydrobenzoic acid, ellagic acid, protocatechuic acid, and epigallocatechin gallate, against that of PECN at 254 nm. Only peaks with a retention time (RT) of 20.14 min in PECN exhibited similar retention time with cinnamic acid.

The HPLC profiles of PECN were measured at six different wavelengths, namely, 210, 254, 280, 300, 330, and 366 nm (Figure 7). At 366 nm, the UV spectra analysis of PECN demonstrated three major peaks labelled as P1 (RT=18.434 min), P2 (RT=18.659 min), and P3 (RT=20.148 min). Further analysis demonstrated that these three major peaks showed their *λ*_max_ values in the region of 222.5-332.6, 192.0-336.2, and 216.6-335.0 nm, respectively, suggesting the presence of phenyl chroman (C_6_-C_3_-C_6_) skeletal structures. As depicted in Figure 8, a comparison between the chromatogram of the standard compounds and the chromatogram of PECN monitored at 254 nm revealed that only peak P3 showed a similar retention time with cinnamic acid indicating the presence of cinnamic acid in PECN.

### 3.6. Phytoconstituents of PECN following the GC-MS Analysis

The GC-MS analysis of PECN resulted in the identification of 242 compounds, whereby the major compounds present at higher percentages were lupeol trimethylsilyl (TMS) ether (4.08%), stigmasta-3,5-diene (3.10%), cyclononasiloxane, octadecamethyl- (2.51%), *γ*-tocopherol, TMS derivative (2.20%), hexasiloxane, tetradecamethyl- (2.19%), stigmast-5-ene, 3*β*-(trimethyl siloxy)-, (24S)- (2.18%), cyclodecasiloxane, eicosamethyl- (1.99%), 2H-1-benzopyran-2-one, 7-(5-butoxy-6-methyl-2H-benzotriazol-2-yl)-3-phenyl- (1.93%), *α*-tocopherol, TMS derivative (1.68%), 1,3-benzenedicarboxylic acid, (4-bromophenyl)sulfonyl]oxy]-, dimethyl ester (1.57%), heptasiloxane, hexadecamethyl- (1.46%), 2-butenedioic acid, 2,3-bis[(2,4-dimethylphenyl)amino]-, and dimethyl ester (1.28%).

## 4. Discussion

Earlier study by Abdul Rahim et al. [9] has demonstrated that the dose-dependent antinociceptive activity of MECN occurs at peripheral and central levels through the activation of opioid receptor system and modulation of the NO-mediated/cGMP-independent pathway while further investigation by Zakaria et al. [10] revealed that the antinociceptive activity of MECN also involved the modulation of protein kinase C-, bradykinin-, TRPV1 receptors-, and glutamatergic-signaling pathways; opioidergic, *α*_2_-noradrenergic, *β*-adrenergic, adenosinergic, dopaminergic, and muscarinic cholinergic receptors' systems; and the opening of various K^+^ channels. As a result of these findings, an attempt was made to partition MECN bioactive compounds according to polarity using petroleum ether, ethyl acetate, and water and testing each partition for their antinociceptive potential. Based on the preliminary investigation using the abdominal writhing test, PECN was found to exert the most effective antinociceptive activity and subjected to further investigation. Further study revealed that the partition also exhibited a dose-dependent antinociceptive activity at peripheral and central levels and this activity occurs through the nonselective opioid receptors activation and modulated via the NO-mediated/cGMP-independent pathway (Zakaria, 2019; personal communication).

In the present study, the mechanisms of antinociception triggered by PECN were further investigated and the results obtained revealed the ability of PECN to inhibit capsaicin-, glutamate-, PMA-, and bradykinin-induced nociceptive response measured using their respective paw licking model. The ability of PECN to induce antinociceptive effect by (i) attenuating capsaicin-induced nociceptive response suggested the partition ability to inhibit the TRPV1 receptors; (ii) attenuating glutamate-induced nociceptive response implied the ability of the partition to inhibit the N-methyl-D-aspartate (NMDA)/non-NMDA receptors or hindered the release of NO/NO-related substance; (iii) attenuating PMA-induced nociceptive response proposed the partition potential to reduce phosphorylation of PKC-activated TRPV1 receptor pathway; and (iv) attenuating bradykinin-induced nociceptive response suggested the ability of the partition to inhibit the activation of B_2_ receptors. Moreover, PECN-induced antinociceptive activity was also demonstrated to be partially reversed after pretreatment with several receptor antagonists, namely, YOH, PDL, HAL, CAF, and ATR, thus, suggesting that the partition's antinociceptive potential involved synergistic modulation of the *α*_2_-adrenergic, *β*-adrenergic, nonselective dopaminergic, adenosinergic, or muscarinic cholinergic receptor systems. Lastly, the antinociceptive activity exerted by PECN was also proven to be influenced by the activation of different types of K^+^ channels. Pretreatment with different inhibitors of K^+^ channels, namely, GLIB, APA, CHAR, and TEA, changed the intensity of antinociceptive activity of PECN, thus, suggesting that the activity was modulated via the activation of ATP-sensitive-, small conductance Ca^2+^-activated-, large conductance Ca^2+^-activated-, and nonselective voltage-dependent-K^+^ channels, respectively. It is worth mentioning that only APA and CHAR caused a full reversal of PECN-induced antinociceptive activity.

The association between TRPV1 receptors, glutamatergic and bradykininergic pathways, and PKC-mediated system in pain transmission has been well acknowledged and is briefly discussed below. The involvement of TRPV1 receptors in the modulation of different modalities of pain sensation and for tissue injury-induced thermal hyperalgesia has been greatly acknowledged. In addition to capsaicin, the TRPV1 receptors are also activated by noxious stimuli such as high temperatures, acidic/low pH, voltage, and anandamide [15]. It is believed that this receptor is inactive at normal body temperature via dynamic regulation and threshold temperature can be reduced at a lower pH or in the presence of capsaicin. Further stimulation of TRPV1 receptors can be achieved by inflammatory mediators such as bradykinin, serotonin, histamine, or prostaglandins, which stimulate TRPV1 receptors among others via the PKC-dependent pathways. Stimulation of TRPV1 receptors by PKC potentiates capsaicin- or proton-induced responses and reduces the temperature threshold for TRPV1 receptors activation inside the cell resulting in neurogenic inflammation [16]. On the other hand, glutamate, a major excitatory neurotransmitter, has been greatly acknowledged to play role in the nociceptive processes with its receptors found in the brain, spinal cord, and periphery that are involved in pain sensation and transmission. Administration of glutamate to the spinal cord or periphery induces nociceptive behaviors whereas inhibition of glutamate receptors or its release at the central or peripheral levels reduces acutely- or chronically-induced pain in animals. The activation of the glutamatergic system in the nociceptive neurotransmission involves the modulation via the NMDA or non-NMDA receptors, NO or NO-related substances discharge, or activation of the PKC pathway [17, 18]. Moreover, PKC, an enzyme localized in the peripheral and central sites that regulate pain, phosphorylates a number of cellular components such as TRPV1, NMDA, glutamate, and bradykinin receptors in the nociceptive signal transduction pathways. PKC induced phosphorylation of TRPV1 receptors results in potentiation of capsaicin- or proton-evoked response and reduction of temperature threshold for TRPV1 receptor activation [19]. Moreover, activation of PKC triggers the TRPV1 receptors to enhance glutamate release leading to increase nociceptive transmission at the first sensory synapse that might contribute to persistent pain conditions [20]. Furthermore, bradykinin has been reported to sensitize peripheral and central nociceptors with actions on the former which triggers the release and synthesis of other second messengers such as prostaglandins, NO, and neurokinins, which cause a reduction in pain threshold [21]. As a potent inflammatory peptide messenger, bradykinin is released from damaged tissues during neurogenic inflammation and acts preferentially at the B_2_ receptors found on various cells including nociceptive primary afferent neurons to directly activate, in part, the PKC-signaling pathway. Bradykinin also acts at the central level via the B_2_ receptor-dependent mechanism and activation of the PKC pathway to potentiate the glutamatergic synaptic transmission to produce pain hypersensitivity [22]. Other than that, bradykinin triggers pain hypersensitivity through the NMDA receptor-dependent mechanisms and potentiated TRPV1‐modulated synaptic responses by activating the PLC‐PKC pathway [23]. This complex interaction between several pathways of nociception might be used to plausibly explain the ability of PECN to attenuate nociceptive response triggered by capsaicin, glutamate, bradykinin, and PMA.

Once the antinociceptive potential of a compound has been established, it is important to elucidate the mechanisms of action involved in the modulation of the observed antinociceptive activity. Using an appropriate pharmacological tool, the mechanisms of action of certain pharmacological activity can be elucidated. For example, studies carried out to establish the mechanism of antinociceptive activity of certain substances utilized various antagonists of different receptors to manipulate their effectiveness to inhibit nociceptive response [24, 25]. Basically, the antinociceptive activity of a compound can be postulated to involve modulation of opioid receptors if its antagonist (i.e., naloxone) attenuates the compound's antinociceptive activity. Receptors respond to noxious stimuli and transmit the information via afferent or sensory fibers to the CNS and are classified into opioid and nonopioid receptors [26]. As described earlier, PECN was found to work via a nonselective opioid receptor system based on the ability of antagonists of *μ*-, *δ*-, and *κ*-opioid receptors to attenuate its antinociceptive activity, respectively [10]. The application of non-opioid-mediated analgesics, other than NSAIDs and acetaminophen, in the treatment of various types of pain, has also been widely acknowledged [27]. Although inhibition of COX activity is thought to be largely responsible for both the therapeutic and adverse effects of this class of drugs, shreds of evidence gathered over the past two decades have demonstrated effects of nonopioids beyond the inhibition of COX and prostaglandin synthesis and include their interaction with monoaminergic (i.e., noradrenergic) [27, 28], dopaminergic [29, 30], adenosinergic [31, 32], and muscarinic cholinergic [33, 34] systems to name a few. In the present study, the ability of PECN to manipulate antinociceptive activity via several nonopioids receptor systems, namely, *α*_2_-adrenergic, *β*-adrenergic, nonselective dopaminergic, adenosinergic, or muscarinic cholinergic, has been proven as indicated by the partial decrease in antinociceptive intensity of PECN when prechallenged with the antagonist of the respective receptor system. Interestingly, the mechanisms of antinociception modulated by acetaminophen, a classic nonopioid analgesic, have been reported to be mediated via the dopaminergic and NO systems. Concurrently, PECN has also been reported in the earlier study to affect the NO-mediated pathway [10]. In addition, several drugs that increase monoamine availability or activate the opioidergic systems have also been reported to show antinociceptive activity via the activation of adenosine receptors [31]. This suggestion corresponds well with PECN, which was shown earlier to exert opioid-mediated antinociceptive activity [10] and in the present study to induce a monoaminergic- and adenosinergic-activated antinociceptive activity.

Interestingly, the ability of PECN to attenuate nociceptive transmission via the modulation of adenosinergic and serotonergic systems as demonstrated in the present study is concurrent with a report made by Luchese et al. [35]. The ability of opioid analgesic such as morphine to exhibit antinociceptive activity via the activation of muscarinic receptors [36] as well as the potential of isopulegol to exert antinociceptive activity via the activation of opioid and muscarinic receptors [37] supports the present observation on the ability of opioid-mediated PECN to exert antinociceptive activity via the activation of muscarinic receptors. Based on the results obtained, it is interesting to highlight that the ability of PECN to exert a non-opioid-mediated antinociceptive activity might provide an answer to the increasing demand for finding new/novel analgesics with potentially fewer side effects associated with the use of opioid analgesics [38].

Another important factor in the modulation of pain signals in the human nervous system is K^+^ ion channels [39–41]. These channels, which are widely distributed in various body cells including neurons and muscles, are membrane proteins that work along with the Na^+^-K^+^ ATP pump to allow rapid and selective flow of K^+^ ions across the cell membrane and, thus, facilitate changes in plasma membrane and intracellular voltage (depolarization and repolarization, generation of action potential, and conductance of electrical impulse) in the neurons. The electrical excitation of peripheral terminals of sensory neurons is the first step in the generation of most pain signals in the mammalian nervous system. These peripheral “nociceptive” signals may be amplified centrally to reach pain-inducing intensity but, nevertheless, the peripheral signal is almost invariably necessary to trigger most types of pain. The excitation of peripheral nociceptive terminal or fiber is brought about by an intricate set of ion channels that are coordinated to produce a degree of excitation that is proportional to the strength of the external stimulation. Several types of K^+^ channels are identified, especially in nociceptors, such as the ATP sensitive K^+^ channels, small conductance Ca^2+^-activated K^+^ channels, large conductance Ca^2+^-activated K^+^ channels, and nonselective voltage-dependent K^+^ channels to name a few. However, in many disease states, this coordination is disrupted resulting in deregulated peripheral excitability which, in turn, may underpin pathological pain states [42]. Due to the fact that channelopathies often underlie pathological pain states, various researches have focused on developing prospective analgesics that target ion channels [43]. Accumulating study related to K^+^ channel physiology has discovered numerous promising leads for the development of novel analgesics. Several K^+^ channel subunits have been recognized to be directly gated to pain-relevant stimuli whereas others are specifically modulated by inflammatory processes. In the present study, PECN-induced antinociceptive activity was fully inhibited upon blocking of the large and small conductance Ca^2+^-activated K^+^ channels whereas blocking of the other K^+^ channels caused only partial inhibition of PECN-induced antinociceptive activity. Generally, the opening of Ca^2+^-activated K^+^ channels, which are found in dorsal root ganglion (DRG), during neuronal firing in response to increases in intracellular Ca^2+^ hyperpolarizes the membrane and provides feedback inhibition that limits Ca^2+^ influx and excitability, making them powerful regulators of synaptic transmission at nerve terminals. One of these channels, the large conductance Ca^2+^-activated K^+^ channels, is thought to influence excitability more prominently and their significance in pain transduction has been proven via a functional coupling with TRPV1 receptors in nociceptors [44]. Moreover, PGE_2_ and other inflammatory mediators reduce large conductance Ca^2+^-activated K^+^ channel activity in nociceptors [45, 46] while deletion of these channels in the neurons enhances inflammatory pain without affecting acute or neuropathic behaviors [45, 47]. Moreover, the small conductance Ca^2+^-activated K^+^ channels, which are also detected in the DRG, may also contribute to pain phenotypes as these channels have been reported to be the downstream targets of NMDA receptor- (NMDAR-) mediated Ca^2+^ influx [45]. This suggestion was based on the observation that removal of the NMDA receptor 1 (NR1) subunit in DRG induces hyperexcitability and pain hypersensitivity, which can be pharmacologically reversed by the inhibitor of small conductance Ca^2+^-activated K^+^ channels [48]. These findings on the involvement of large and small conductance Ca^2+^-activated K^+^ channels in the modulation of pain processes and the ability of the respective channels' blocker to completely inhibit the PECN-induced antinociceptive activity when assessed using the peripherally-modulated nociceptive model (abdominal writhing test) suggested that both types of Ca^2+^-activated K^+^ channels modulate the antinociceptive activity of PECN, at least, at the peripheral level. In addition to those subtypes of K^+^ channels, the ATP sensitive K^+^ channels and nonselective voltage-dependent K^+^ channels have also been found to play roles in the transmission of nociceptive response and might explain their partial reversal effect on the peripherally-mediated antinociceptive activity of PECN. The ATP-sensitive K^+^ channels, which are also expressed in the subpopulation of DRG neurons, are inhibited by intracellular ATP and, thus, were suggested to play a protective role in neurons under pathological conditions. Interestingly, the enhancers of ATP-sensitive K^+^ channels have been shown to modulate nociceptive transmission by causing the nociceptive neurons hyperpolarization as well as decreasing the bradykinin-induced pain intensity [49]. The voltage-gated K^+^ channels, which are also found predominantly in the small DRG neurons, have been shown to be expressed in nociceptors. Several families of voltage-gated K^+^ channels such as the Kv4 and Kv7 channels have been proven to play a role in nociceptive processes [50, 51]. For example, the Kv4 subunits have been associated with pain plasticity [50] whereas the Kv7 subunits have been reported to play a role in inflammatory pain [52]. These reports support the present findings on the potential of both types of K^+^ channel to partially affect the intensity of the antinociceptive activity of PECN at the peripheral level. According to Tasantoulas and McMahon [45], multiple K^+^ channel deficiencies represent a general source of overexcitability within the peripheral pain pathways. Furthermore, activation of a K^+^ current in most neurons is likely to provide an antiexcitatory effect regardless of the source of overexcitability. Therefore, it is plausible to suggest that pharmacological K^+^ channel enhancement could be utilized as a strategy for the management of pain. Interestingly, several of the well-known NSAIDs such as diclofenac and celecoxib have been reported to possess strong K^+^ channel opener activity, which is strongly suggested to partly contribute to their analgesic efficacy [53, 54]. Based on these reports, it is plausible to propose that PECN also possesses the K^+^ channel opener activity. It is also worth mentioning that the involvement of different K^+^ channels in the modulation of PECN-induced antinociceptive activity was concurrent with reports on diclofenac and docosahexaenoic acid [55, 56]. The ability of PECN to activate different types of K^+^ channels could be attributed to the presence of volatile and nonvolatile bioactive compounds, as well as phenolics/flavonoids-based bioactive compounds. These compounds were believed to act either individually or synergistically leading to the opening and activation of these channels.

With regards to the phytoconstituents of PECN, phytochemical screening of the fraction demonstrated the presence of several classes of bioactive compounds, namely, flavonoids, saponins, and triterpenes. Basically, these classes of bioactive compounds have been reported to possess antinociceptive effects [57–62]. Further analysis using the HPLC procedure revealed the presence of a small number of bioactive compounds of the phenolic-based and comparison of the chromatogram obtained for PECN against that of several phenolic compounds demonstrated the presence of at least cinnamic acid. Interestingly, a recent study had revealed the potential of cinnamic acid to alleviate pain [63]; thus, it might help to support the present observations. In addition, GC-MS analysis of PECN revealed the presence of trimethylsilyl (TMS) derivatives of *γ*-tocopherol, *α*-tocopherol, and lupeol of which *α*-tocopherol [64] and lupeol [65] have been shown to exert antinociceptive activity whereas *γ*-tocopherol has been reported to enhance the anti-inflammatory potential of acetylsalicylic acid [66]. These bioactive compounds might also contribute to the observed antinociceptive activity of PECN. In conclusion, the present study has demonstrated the antinociceptive activity of PECN to occur through the modulation of a number of nociceptive pathways (i.e., PKC-, bradykinin-, glutamate-, and TRPV1-mediated) and partial inhibition of several nonopioidergic systems (i.e., serotonergic, *α*_2_-adrenergic, nonselective dopaminergic, adenosinergic, or muscarinic cholinergic). Moreover, the antinociceptive activity of PECN is fully inhibited if the small and large conductance Ca^2+^-activated K^+^ channels were blocked, but it is partially inhibited if the ATP-sensitive and nonselective voltage-dependent K^+^ channels were blocked. The occurrence of antinociceptive activity via multiple mechanisms is possibly due to the individual or synergistic action of volatile and nonvolatile bioactive compounds present in the fraction. Further investigations focusing on isolating the possible bioactive compounds from PECN are warranted and currently being planned.

## Data Availability

The supporting materials can be obtained upon request via email to the corresponding author.
